# Supplementary material for: Curated and harmonised transcriptomics datasets of interstitial lung diseases
Source: Data Brief. 2025 Oct 14;63:112139. doi: 10.1016/j.dib.2025.112139 (PMC12581653; doi:10.1016/j.dib.2025.112139)
Supplement: Supplementary file 1 [file mmc1.zip › Supplementary_material/RNA-seq/fastQC reports/GSE169500/SRR14057116_fastqc.html]

SRR14057116.fastq.gz FastQC Report 

FastQC Report

Mon 5 Feb 2024  
SRR14057116.fastq.gz

## Summary

- Basic Statistics
- Per base sequence quality
- Per sequence quality scores
- Per base sequence content
- Per sequence GC content
- Per base N content
- Sequence Length Distribution
- Sequence Duplication Levels
- Overrepresented sequences
- Adapter Content

## Basic Statistics

| Measure | Value |
| --- | --- |
| Filename | SRR14057116.fastq.gz |
| File type | Conventional base calls |
| Encoding | Sanger / Illumina 1.9 |
| Total Sequences | 12594091 |
| Sequences flagged as poor quality | 0 |
| Sequence length | 25-368 |
| %GC | 50 |

## Per base sequence quality

## Per sequence quality scores

## Per base sequence content

## Per sequence GC content

## Per base N content

## Sequence Length Distribution

## Sequence Duplication Levels

## Overrepresented sequences

| Sequence | Count | Percentage | Possible Source |
| --- | --- | --- | --- |
| AAGCTCAGGGAGAGCCCTGTTAGGGCCGCCTCTGGCCCTAGTCTCAGACC | 202596 | 1.6086591719878789 | No Hit |
| GGGCCACGAGCTGAGTGCGTCCTGTCACTCCACTCCCATGTCCCTTGGGA | 195634 | 1.5533792792191197 | No Hit |
| GGGAATGTCTACGTGCGTATGCACGTGGCACTCTCTGCCCGAGGTCCGGG | 130958 | 1.039836856824363 | No Hit |
| ACACTGAGGACTCTGTTCCTCTCCTTTCCGCCTAGGGGAAAGTCCCCGGA | 98963 | 0.7857891450839921 | No Hit |
| AGAGCATGACCGATGGATTCCAGTTCGAGTATGGCGGCCAGGGCTCCGAC | 89216 | 0.7083957071613981 | No Hit |
| GCTGCTACAGGAGAATAGCAGACAGGTATAGTTAAGAAACAACTTTATTT | 76576 | 0.6080311790664368 | No Hit |
| ACACTGAGGACTCTGTTCCTCCCCTTTCCGCCTAGGGGAAAGTCCCCGGA | 74460 | 0.5912296488885144 | No Hit |
| CAATATCAAGGAAGGGCAGGCGTGATGGCTTATTTGTTTTGTATTCAATG | 72845 | 0.5784061747687864 | No Hit |
| ATGTTCTGGGAGGCCTCGGTGGACATCAGGCGCAGGAAGGTCAGCTGGAT | 58482 | 0.46436062753556406 | No Hit |
| TTTACACCTGCTAGGGTGTTCAAAGGTCAGTGCTATAGAAATTCAGTATC | 56625 | 0.4496156173557901 | No Hit |
| CTAAGTGCTGTGTTGTCGTTCCCCCTGCTTAAAATAAAGTTGTTTCTTAA | 51361 | 0.40781823793396443 | No Hit |
| ATTCTTCCTACCCATGAGCATGGAATGTTCTTCCATTTGTTTGTATCCTC | 47711 | 0.3788363924002137 | No Hit |
| TTCTTGTAGATGGCTGCTCTAAAAAGACAAATGAATGGGGAAAGACAATC | 47688 | 0.3786537670721928 | No Hit |
| ACCAATGTCATAGGGTGCAATATCTACAATAGGTAGTCTCACAGCCTTGC | 41923 | 0.33287833159217284 | No Hit |
| GGCCACGAGCTGAGTGCGTCCTGTCACTCCACTCCCATGTCCCTTGGGAA | 38181 | 0.3031659847463386 | No Hit |
| TCTGCAATGCAATCACAATGCCCAAACTAGACCTGCCATTTCTCACACAC | 35452 | 0.2814970925650767 | No Hit |
| AAAGCCAAGAAAACCAACGATGCCAGATACTGAATTTCTATAGCACTGAC | 34707 | 0.27558161998353037 | No Hit |
| ACCCGCAGCTTCTGCTTCTCAGTCAGAAGGTTGTTGTCCTCATCCCTCTC | 28878 | 0.22929800967771313 | No Hit |
| GAAGACGTACCTTTGTTGGAAGACATACCTTTGTTGGAAGACGTACCTTT | 28065 | 0.22284260134375716 | No Hit |
| ATGTTCTGGGAGCCTCGGTGGACATCAGGCGCAGGAAGGTCAGCTGGATG | 26900 | 0.21359223146791617 | No Hit |
| TGTTGTGCTAAACGCTGGGAGGGTACAAGTTGGTCATTCCTAAATCTGTG | 26565 | 0.21093225386413358 | No Hit |
| TTTTTGTTGCCTGTAAAGTGCTCTGGCATTGCCTGAGGATAGATGAGAAA | 26365 | 0.2093442075335171 | No Hit |
| TGGTTGCACGAAACACACTGGGGAATGGAGCAAAACAGTCTTTGAATATC | 23962 | 0.19026383087116014 | No Hit |
| TTCCCCCTGCGCATGCGGGACTGGCTCAAGAACGTCCTGGTCACCCTGTA | 23739 | 0.18849315921252277 | No Hit |
| TCTTCAAGGAGAACTACAAACCACTGCTCAAGGAAATAAAAGAGGATACA | 22722 | 0.18041794362133798 | No Hit |
| CAGCATGATTTATAGTCCTTTGGGTATATACCCAGTAATGGGATGGCTGG | 22659 | 0.17991770902719378 | No Hit |
| AGTGTGGCGATTCCTCAGGGATCTAGAACTAGAAATACCATTGACCCAGC | 22343 | 0.17740859582481974 | No Hit |
| AGCGGTTCCGCTGCCCTGAGGCACTCTTCCAGCCTTCCTTCCTGGGCATG | 20883 | 0.16581585761131948 | No Hit |
| TCACACTTCATGATGGAGTTGAAGGTAGTTTCGTGGATGCCACAGGACTC | 19834 | 0.15748655460723604 | No Hit |
| CAGTAGCACCAACGTTGATGAGGTCGTGAGTCAGGGGTCCTAGGAAGCTC | 18725 | 0.1486808377039677 | No Hit |
| ATCAAATTAATGTCTTCCAGCCTACTTGTGTCTTCCAACAAAGGTACGTC | 17519 | 0.13910491833035032 | No Hit |
| TGGTTCCTGGCAAGCCCATGTGTGTTGAGAGCTTCTCAGACTATCCACCT | 17491 | 0.138882591844064 | No Hit |
| TAGGACACTTCTGCCAAAGGAACTCGTGCACAGCACCACAGAACATGGCA | 17478 | 0.13877936883257394 | No Hit |
| TCTTCCAGCCTACTTGTGTCTTCCAACAAAGGTACGTCTTCCAACAAAGG | 17068 | 0.13552387385481018 | No Hit |
| TGTTCCCTCTCCTCATGAGATTGGTGAAGAAAGTATTTGGCAAAGTTCTT | 16927 | 0.13440430119172556 | No Hit |
| ACACCCACCGCAACTGTCTGTCTCATATCACGAACAGCAAAGCGACCCAA | 15916 | 0.12637672699045926 | No Hit |
| GGTTATAATCATTGGCAATGTTGTAATCAACAACCCATGTTATTTCTACA | 15268 | 0.12123145687926186 | No Hit |
| TGACTGGTGATGCTGATATTGGAGTGCATCTGAGGCTGTTGTAGAAATAA | 14955 | 0.11874616437184708 | No Hit |
| TCTTCAAGGAGAACTACAAACCACTGCTCAATGAAATAAAAGAGGATACA | 14147 | 0.11233045719615653 | No Hit |
| CAACCCATACAACCAACAGGCTGCGTACACTACCAAGGAAGCTGCTGTGT | 14123 | 0.11213989163648254 | No Hit |
| TGCGAAAAGTTACTTTCAATGGACTGAACCAGATGATTGTCATAGAACTG | 14091 | 0.1118858042235839 | No Hit |
| TCTTTCTGGCCTGGAGGCTATCCAGCGTACTCCAAAGATTCAGGTTTACT | 13954 | 0.11079799248711161 | No Hit |
| CAGAAGCTCTTTATGTCAAACTTCTCAAGTTGATTGACCTCCAGGTAATA | 13932 | 0.1106233073907438 | No Hit |
| TTTCCTCATGGGGACCCAGTGTGCGATGGCTGCACACAGCAGCTTCCTTG | 13909 | 0.1104406820627229 | No Hit |
| AAGAGAAAGCAGTGCAAACCTTCCCGTGGCCGCAAGCGTGGCATCTGCTG | 13720 | 0.10893997828029034 | No Hit |
| GGAATGTCTACGTGCGTATGCACGTGGCACTCTCTGCCCGAGGTCCGGGG | 13590 | 0.10790774816538963 | No Hit |
| ATTTTCCCAGGTGGGGCCTGCCTGGGGAAAGCTTGTGGCCGGAAGAGAAA | 13443 | 0.1067405341123865 | No Hit |
| TCAACGTACTCCATGCCTGGCAGCTTCATCCCGTACTTGTCCACGCACCA | 13281 | 0.10545421658458716 | No Hit |
| TCAGAAAGGTCACGTCCTGCTCTTGGTCCTTGTCAGTGCCATGTTCTGTG | 13057 | 0.10367560469429671 | No Hit |
| AAAACAGCGTCTTACTGGGGAGGGATATGTGCTTTCTCATCTATCCTCAG | 12887 | 0.1023257653132727 | No Hit |
| TGTGCCCTGGAAGCGGAATGGAAACTTGCAGGGCTGTCCTTCAGCGTTGC | 12620 | 0.10020572346189972 | No Hit |

## Adapter Content

Produced by FastQC (version 0.11.7)
